# The hidden professionalism curriculum: Teach it, see it, do it and repeat!

**DOI:** 10.12688/mep.20276.1

**Published:** 2024-05-28

**Authors:** Scott W. Oliver, Kathleen Collins

**Affiliations:** 1 Medical Education Department, NHS Lanarkshire, Bothwell, Scotland, G71 8BB, UK; 2 School of Medicine, University of Glasgow, Glasgow, Scotland, G12 8QQ, UK; 3 UK Council for Educators of Medical Professionalism, Glasgow, UK; 4 NHS Education for Scotland, Glasgow, UK

**Keywords:** Professionalism, Undergraduate, Teaching, Hidden Curriculum, Learning, Pedagogy

## Abstract

**Background:**

Professionalism is a complex and multifaceted component of medical education. Historically, students have learned about professionalism informally and as part of the hidden curriculum. Currently, professionalism is increasingly prominent in formal curricula, but uncertainty remains regarding optimal professionalism pedagogies. In this study, the authors explored medical students’ exposure to professional topics and considered factors that enabled students to correctly recognize and manage these issues.

**Methods:**

Convenience sampling was used to recruit medical students from existing clinical attachments at the authors’ hospital. A semi-structured interview format was used to explore participants’ awareness of professional issues within fictional vignettes created using published regulatory guidance. The interview transcripts and interview guide field notes were then analyzed.

**Results:**

The data suggest that students require a combination of didactic teaching and experiential learning to reliably recognize and manage professional issues. Didactic teaching alone enabled topic recognition, but with uncertainty about management strategies. Experiential learning alone led to erratic recognition of the subject and reliance upon role modeling to guide its management. This work stimulates faculty development to enhance teaching professionalism.

**Conclusions:**

Undergraduate medical education on professionalism must be introduced into the formal curriculum. Didactic teaching is required to scaffold experiential learning. Failure to do so renders students unable to reliably recognize or manage professional issues encountered in clinical practice. Further research questions were identified to progress this work.

## Introduction

Health care professionalism is a complex and nuanced topic. While no consensus definition currently exists, the literature consistently proposes language that includes adherence to rules, perceptions, and expectations of others, and behaviors of the individual ^1^. This study draws on a decade of experience in teaching professionalism to undergraduate and postgraduate healthcare students. It defines ‘professionalism’ as behaviors, attributes, and actions exhibited and upheld by a healthcare professional, which results in good clinical practice and facilitates trusting relationships with patients and caregivers.

Many authors have called for more formal teaching of professionalism ^2, 3^. This is partly driven by faculty perceptions of medicine becoming de-professionalized, ongoing consistent reports of unprofessional behavior in the workplace ^4^, and ever changing societal and cultural expectations. More broadly, healthcare professionalism is essential to public trust. Lapses in professionalism are a stimulus for regulatory referrals that ultimately result in doctors being disciplined, suspended from practice, or erased from the medical register ^5^.

The literature describes many examples of ‘poor professionalism’ and its impact and complexities in healthcare ^2, 6– 8^. Although work has been undertaken to understand how medical students learn professionalism ^9, 10^, this literature is comparatively sparse. Professionalism teaching is often consigned to the hidden curriculum, although in recent years, topics aligned to ‘professionalism’ have begun to emerge in formal curricula. Published work suggests that clinical context and experiential learning are key elements of teaching professionalism ^10, 11^. However, medical schools have limited control over clinical learning environments with respect to professional behaviors. There are reports of student distress in the face of dissonance created by exposure to real-life clinical attachments, in comparison with sterile descriptions of idealized professional practice ^12^. Developing and refining professional pedagogies seems to be key to advancing this important aspect of undergraduate medical education.

Most authors have investigated students’ experience of learning professionalism from an institutional perspective, typically involving single schools with a single formal and hidden curriculum. In this study, the authors explored professionalism learning from the perspectives of medical students from three Scottish medical schools during their attachment to a single district general hospital, where the authors worked as clinical teaching fellows. A single high-level curriculum document defined the formal curriculum for all three schools ^13^, but each school differed in practical implementation. In effect, the students had the same formal curriculum exposure, but differential exposure to the hidden curriculum. The purpose of this study was to investigate students’ respective exposure to professionalism issues and their ability to recognize and manage these situations, and to compare the findings between student cohorts from different medical schools. This paper reports these findings, their integration into a series of ‘teaching professionalism’ faculty development workshops, and the resulting insights into how professionalism pedagogies at medical schools might be optimized.

## Methods

A series of eight fictional clinical vignettes were developed with reference to the four domains of Good Medical Practice ^14^ (see Extended Data ^15^). A semi-structured interview guide (see Extended Data ^15^) was designed to establish participants’ ability to recognize and action aspects of professionalism within each vignette, their prior exposure to these issues, and the extent to which this had arisen in the formal, informal, or hidden curriculum.

A convenience sample of participants was recruited from undergraduate medical students with clinical attachment within the authors’ district general hospital. Written study information was provided for each block induction, followed by a single email invitation sent by an undergraduate administrator. Each author recruited participants from the other author’s student cohort to limit potential conflicts of interest. Approximately 150 students were invited to participate over a 12 month period.

The interviews were conducted at a private office within the hospital. These were timetabled during the working day, but outside the students’ scheduled clinical placement activities. Both authors participated in the interviews. Field notes were taken using interview guides and audio recordings were transcribed for analysis. A random number generator ( www.random.org) was used to determine which three vignettes from the bank of eight vignettes were discussed with each participant. This reduced the potential for participants to have heard of the vignettes from earlier participants before their own interviews.

Emergent probing ^16, 17^ and advocacy with inquiry ^18^ were utilised to explore relevant topics as they arose during the interviews. Constant comparison ^19^ was used between interviews to tailor the interview strategy, and interviews were conducted until data saturation ^20, 21^ was reached. Both authors independently analyzed the interview transcripts, with differences in coding resolved by consensus ^22^.

Thematic analysis findings were used to create a faculty development workshop: The hidden professionalism curriculum. The workshop considered how undergraduate medical students might learn about professionalism and how it might, therefore, be better taught.

Ethical approval for this study was granted by the University of Dundee Research Ethics Committee (reference UREC15078, 22 June 2015). Written informed consent was obtained from each participant before the interviews began.

## Results

Thirteen students (seven male and six female) from three medical schools participated in the study. One participant had a previous undergraduate degree and medicine was the first degree for the remaining 12 participants. Three participants were registered at medical school 1, four from medical school 2, and six from medical school 3. This broadly reflects the total proportion of students attached to hospitals from each institution across the academic year.

Three major themes were identified through thematic analysis:

1.   Recognition: Participants showed difficulty in recognizing professionalism issues within each vignette unless they had previously been explicitly taught it;

2.   Experiential learning and role modelling: Exposure to either strongly influenced participants’ approach to professionalism issues, and the degree to which their response was flexible according to context.

3.   Learning resource awareness: Participants cited a very limited repertoire of learning opportunities and resources despite the broad scope of the topics being discussed.

### 1. Recognition

It was apparent that when a student reported having no formal teaching or experiential learning on a given topic, they were unable to recognize it within the vignettes. It was striking across the interviews that participants were unable to identify or define significant topics, such as probity, teamwork, and the influence of human factors.

Topics that could be identified had previously been taught during earlier clinical attachments and university sessions. Recognizable topics tended to be those considered more obvious (e.g., consent, confidentiality, racism, bullying):

*“If I had met the patient, I would like to check and ask questions to the patient about sharing information...I would know that patients have right, and confidentiality, even if it is their next of kin, so if they have capacity then we need to ask for permission whether they are happy...We have heard of this in lessons [and] small group teaching in 1 ^st^ and 2 ^nd^ year...”* (Participant 1)

*“Well obviously they shouldn’t be making racist remarks whilst they are working, but also that the team wouldn’t work well together if there are racist remarks.”* (Participant 2).

Subtle issues (e.g., duty of candor, human factors, contractual considerations) were repeatedly missed even when prompts were given. When asked whether they thought it was reasonable to document in casenotes on behalf of a colleague, Participant 1 responded, *“Am I the only other doctor in the team? Then I would undertake the request...”.* Having not had any formal teaching about medical record keeping, they assumed that this was related to staff shortages, as this was the context where they had witnessed a similar situation previously.

### 2. Experiential learning and role modelling

When participants recognized a professionalism issue, their suggested management of the situation was related to their previous experiences of that issue. If they had witnessed a senior colleague managing a similar scenario, the student reported that the colleague’s approach was correct, although this was not always the case. Without having witnessed a scenario actioned in a real clinical setting, participants struggled to explain how they should manage this situation.

*“...it is kind of an assumed thing that we are not going to do that. I mean there are talks about confidentiality, about ‘don’t talk about patients in public’...I think things like cultural sensitivity, making inappropriate comments, people are guilty of it, but I think it's taken as a presumption that you’re not going to...It’s a difficult one; I don’t think we’ve ever had formal teaching on it as such.”* (Participant 11)

***“Interviewer:** Have you heard of people reporting this type of event, and what they overheard or witnessed?*

***Participant**: Can’t think of any examples, no.*

***Interviewer: ** Ok, if you were the person who overheard these comments – what would you do?*

***Participant**: I would probably want to say at the time ... that you would want them to stop talking about it, that it was slightly inappropriate, and you would prefer them not to do it in public, remind them politely, I guess. It's not best to talk about patients and laugh about patients particularly if it's something cultural, but I wouldn’t want to start a major confrontation about it, I’m not the kind of person who likes confrontation. I would maybe discuss it with someone higher up than me, especially if wasn’t the first time.”* (Participant 11)

When the issues within a vignette were correctly identified and actioned appropriately, participants reported having prior explicit teaching on the topic, observed a senior colleague managing it in a real or simulated context, and had personal opportunities to practise their learning in a real or simulated clinical context.

### 3. Resource signposting

The participants were universally uncertain about where they could learn more about professionalism. Moreover, they struggled to identify relevant teaching within their curricula unless this was explicitly labelled as 'professionalism.’ When pushed to suggest additional learning resources, it was typical for students to suggest visiting the websites of large, well-known organizations such as the General Medical Council or generic ‘medical defence organizations.’ They were uncertain about the information these websites might contain, and had not previously accessed any specific documents or resources on these topics.

Notably, no participants suggested that asking a senior colleague for guidance would be a suitable learning resource in the context of developing their knowledge of professionalism. The most striking observation was that almost every participant approached the researchers following the interview to request further teaching about topics introduced during the interview. This was often in the context of having discussed their interviews with non-participant student colleagues, prompting the group to make collective requests for this teaching.

## Discussion

This pilot study sought to explore undergraduate medical students’ learning of professionalism. Interviews were conducted with students from three medical schools that followed the same high-level curriculum, and were implemented slightly differently. The data suggest that students require explicitly labelled didactic teaching alongside experiential learning to reliably identify and manage professional situations. Prior didactic teaching alone enabled the identification of the topic at hand, but its practical management was not necessarily plausible or correct. Prior experiential learning that was unsupported by didactic teaching led to unreliable recognition of professionalism issues, and role modeling of previous encounters guided the management plan. Notably, prior experiential learning arose in the hidden curriculum rather than the controlled curricular space. Without explicitly labelled didactic teaching or experiential learning, students could not recognize professional topics or suggest how they could be tackled in practice.

**Figure 1.**
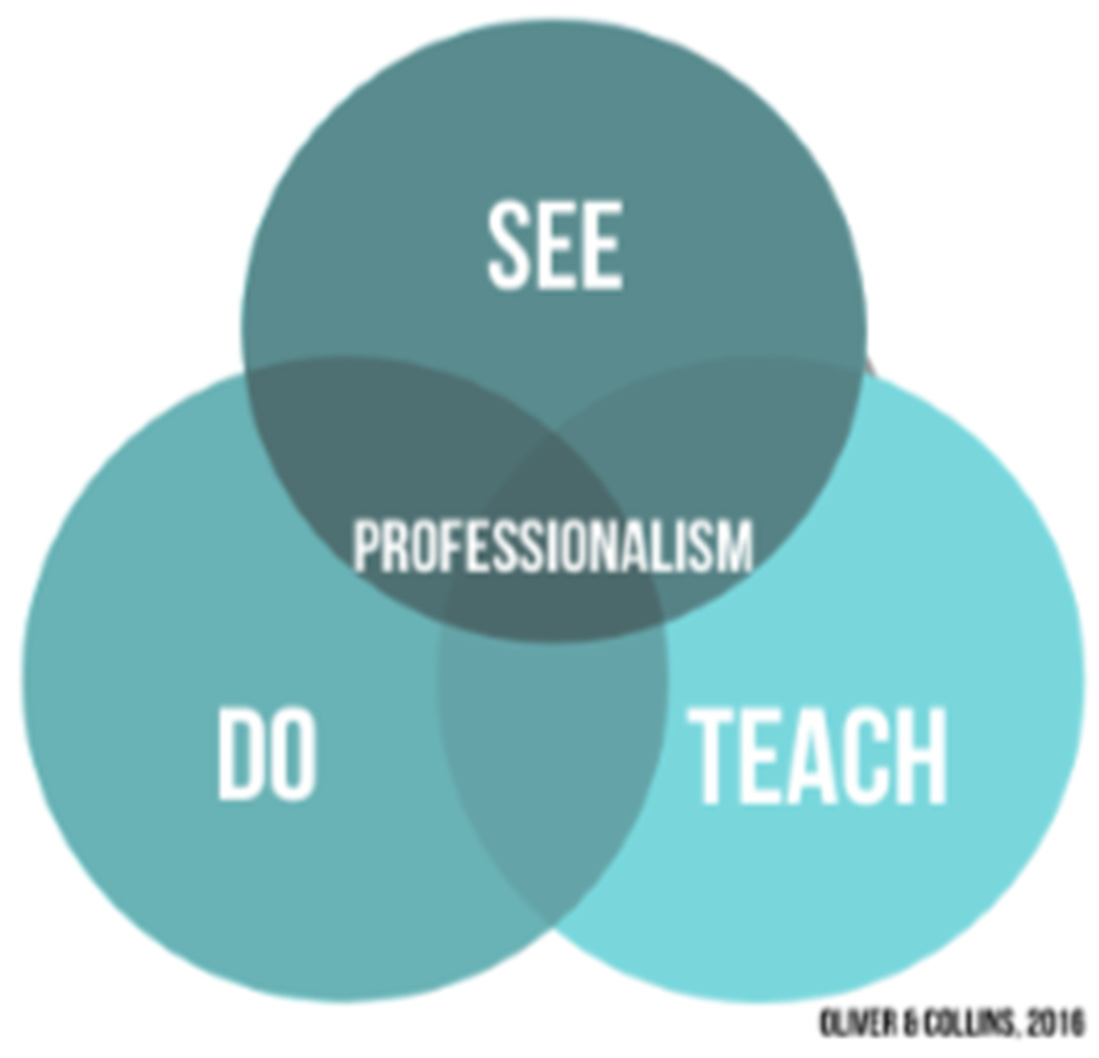
Proposed triad model of professionalism learning.

Therefore, the authors suggest that students require both didactic teaching and experiential learning to safely navigate the clinical landscape from a professional perspective, a conclusion that has also been supported in past literature ^23^. This included being able to recognize the nuances of important topics, such as maintaining confidentiality, probity, and working within clinical teams. Moreover, this learning should be labelled according to topics and features within the formal curriculum. In keeping with Kolbs and experiential learning theories ^24^, students also require time to reflect and experiment in safe spaces with respect to each topic. It is suggested that a triad of professional learning ( Figure 1) is required, with component parts delivered consistently across the undergraduate curriculum. Didactic teaching does not necessarily have to precede experiential learning as long as both are offered in sufficient quantities.

Adopting this approach may require institutions to radically rethink the existing strategies for teaching professionalism. There are, however, some simple steps that could facilitate enhanced professionalism learning across existing programs of pre-clinical and clinical training. Trusted organizations, such as the General Medical Council, have already published resources on professionalism topics ^25, 26^. Timetable redesign can deliberately expose students to consent, capacity assessment, and confidentiality issues during their existing attachments. Snapshot factsheets and similar can be signposted to students during their clinical blocks, for example a ‘consent’ factsheet during a pre-operative surgical ward round.

The authors acknowledge the several limitations of this study. The vignettes used for participant interviews may have been too artificial and subtle to facilitate the recognition and discussion of the professionalism issues contained within them. Constraints including time, funding, and participant availability limited efforts to validate the vignettes. Future work should include the development of scenarios that are validated in terms of authenticity and content. Formal curriculum mapping of participants’ earlier exposure to topics within the vignettes could provide additional insights, for example, when this content could be most effectively taught within the curriculum. While data saturation was reached in this pilot study, a larger number of students from a broader range of host institutions would be required to test the hypotheses generated by this work and those described above. The original data collection for this study was performed before the Covid-19 pandemic; curricular redesign, which could potentially influence current student learning about professionalism in ways that were unaccounted for by this study, and future research should consider this question.

It is notable that participant recruitment to this study was slow, a phenomenon the authors and colleagues have anecdotally noted in other areas of undergraduate professionalism education research. Further work should explore this concept in more detail. The optimal nature, setting, and content of experiential learning about professionalism and its juxtaposition with didactic teaching also deserve further study. This is especially relevant when considering the logistical arrangements needed to facilitate large-group teaching during geographically distributed clinical attachment. Perhaps there is a role for clinical simulation courses in providing this learning space.

This pilot study generated interesting insights that provide a substrate for future research and stimulate discussions about local curricular change. It seems there is an urgent need to formalize professional teaching in medical schools. It cannot be presumed that students simply absorbed professionalism as part of the hidden curriculum. This assumption is unfair to students, their future employers, and their patients. Strategic changes at the institutional level are required to achieve this goal. However, the faculty development activities prompted by this work have revealed various straightforward opportunities to enhance professional teaching during local clinical attachments. These are anticipated to improve learning about professionalism, foster good clinical practice, and trusting relationships with patients.

## Ethics and consent

Ethical approval for this study was granted by the University of Dundee Research Ethics Committee (reference UREC15078, 22 June 2015). Written informed consent was obtained from each participant before the interviews began.

## Data Availability

The participant information leaflet stated that interview transcripts would remain confidential and would not be shared. This was approved by the institutional ethical review board (University of Dundee, reference UREC15078, 22 June 2015). If a reader or reviewer wishes to obtain access to the interview transcripts they are invited to contact the corresponding author, who in turn will apply for approval from the institutional ethical review board before any data can be shared. Figshare: Interview guides and vignettes. https://doi.org/10.6084/m9.figshare.25368574 ^15^. Data are available under the terms of the Creative Commons Attribution 4.0 International license (CC-BY 4.0).

## About the authors

The authors/interviewers were identified as male and female, respectively. Both were experienced clinicians at the time of the study. Both had completed qualitative research work in prior roles and were in the process of completing formal medical education qualifications (PG Cert and PG Dip Med Ed respectively) and one author (SO) was undertaking a PhD in qualitative research at the time.
