# Supplementary material for: The hidden professionalism curriculum: Teach it, see it, do it and repeat!
Source: MedEdPublish (2016). 2024 May 28;14:39. [Version 1] doi: 10.12688/mep.20276.1 (PMC11195607; doi:10.12688/mep.20276.1)
Supplement: Peer review history

# Reviewer report: Reviewer response for version 1

**DOI:** 10.21956/mep.21706.r37284

**Published:** 2024-06-18

**Authors:** Neil Harrison

**Affiliations:** 1 University of Dundee, Dundee, Scotland, UK

Thank you for this well written, topical and practically helpful study and manuscript.

 The manuscript describes a qualitative study, utilizing semi-structured interviews to consider the impact of formal and informal professionalism teaching on undergraduate medical students.  Uniquely, the study samples students across three medical schools.

 The manuscript is well written but I wonder if the authors might consider the following points:

 - The research question being addressed is not entirely clear.  If this was explicit early in the manuscript it would help the reader navigate the 'story' within the text.

 - The introduction draws on literature from undergraduate medical education.  Do the authors feel that literature from other health professions may provide useful insight?  If relevant literature from other professions is not available, then this in itself would be a helpful statement.

 - The novel aspect of considering three different medical schools is perhaps a little confusing in places.  In the last paragraph of the introduction, the authors identify the differences in these three groups but then state the the 'students had the same formal curriculum exposure'.  I doubt this is the case and in the conclusion you comment in the limitations section that it would have been helpful to understand their different prior formal teaching.  I think you need to be clearer that all three groups are likely to have had different formal teaching and this is indeed a strength of the work.

 - Related to this point about the three different schools, there seems to be a tension between the introduction where three different schools are presented as a strength but then in the results section they are treated as one group.  I don't see this as a problem but think how you are treating/viewing the groups just needs to be clearer.  If you do see the three schools as different enough to be treated as three separate samples, it does also make me wonder about the conclusion that saturation was achieved with such small numbers.  You acknowledge small student numbers in your limitations but I wonder if how you view saturation and the number of students in each group needs to be mentioned in the results section.

 - I wasn't entirely clear on the place of the faculty development workshop.  I don't think this fits in the methods or results section.  From what I understand this was not part of the study but occurred as a result of needs identified by this work and is a suggestion for others to consider following the work.

  I hope these comments are helpful.

Have any limitations of the research been acknowledged?

Yes

Is the study design appropriate and does the work have academic merit?

Yes

Is the work clearly and accurately presented and does it cite the current literature?

Yes

If applicable, is the statistical analysis and its interpretation appropriate?

Not applicable

Are all the source data underlying the results available to ensure full reproducibility?

Yes

Are the conclusions drawn adequately supported by the results?

Partly

Are sufficient details of methods and analysis provided to allow replication by others?

Yes

Reviewer Expertise:

UG medical education, simulation-based education, clinical skills assessment

I confirm that I have read this submission and believe that I have an appropriate level of expertise to confirm that it is of an acceptable scientific standard, however I have significant reservations, as outlined above.

# Reviewer report: Reviewer response for version 1

**DOI:** 10.21956/mep.21706.r37283

**Published:** 2024-06-17

**Authors:** John Frain

**Affiliations:** 1 University of Nottingham, Nottingham, England, UK

The study is relevant to the needs of medical educators.  Sample selection is clear and the participants are representative of the study population.  Together with the extended data, the methodology is clear and appropriate.  The topic guide for the interviews is quite' loose' but specific enough to obtain relevant data.  More information on the coding and analysis of the interviews would have been helpful in terms of reproducibility.  In terms of the results, the reality and impact of the hidden curriculum for medical students is acknowledged.  Students place a value on didactic teaching in aiding recognition of professionalism issues and developing the skills to navigate these.  The study highlights absence of an explicit professionalism theme in teaching leaves the potential vacuum of the hidden curriculum where the suboptimal and inappropriate behaviours of seniors may lead to poor role-modelling and adoption of unprofessional behaviours by students.  Several of the vignettes involve scenarios now covered by the GMC's requirement in *Good Medical Practice *(2024) to be an active byststander.  Again, the study should encourage educators to embed teaching of these themes and skills into teaching throughout the curriculum.  It was pleasing to see several of the scenarios also relate professional values to quality of clinical outcome.  The study acknowledges the need to move concepts of professionalism beyond a series of rules and rather a set of skills which aids and augments high quality clinical practice.  This facilitates student engagement with what can seem to them a dry or even unnecessary educational theme.  Overall, the pilot study results provide a good staging post for where many of us find ourselves at the moment as educators in 'professionalism'.

Have any limitations of the research been acknowledged?

Yes

Is the study design appropriate and does the work have academic merit?

Yes

Is the work clearly and accurately presented and does it cite the current literature?

Yes

If applicable, is the statistical analysis and its interpretation appropriate?

Not applicable

Are all the source data underlying the results available to ensure full reproducibility?

Yes

Are the conclusions drawn adequately supported by the results?

Yes

Are sufficient details of methods and analysis provided to allow replication by others?

Partly

Reviewer Expertise:

Evidence based clinical communication and physical examination.  Co-editor of ABCs of Clinical Professionalism (2018), Clinical Resilience (2021) and Equality, Diversity and Inclusion in Healthcare (2023)

I confirm that I have read this submission and believe that I have an appropriate level of expertise to confirm that it is of an acceptable scientific standard.

## References

[1] : The hidden professionalism curriculum: Teach it, see it, do it and repeat!. *MedEdPublish* .2024;14: 10.12688/mep.20276.1 10.12688/mep.20276.1 PMC1119560738915772

# Reviewer report: Reviewer response for version 1

**DOI:** 10.21956/mep.21706.r37287

**Published:** 2024-06-06

**Authors:** Claire Stocker

**Affiliations:** 1 Aston Medical School, Aston University, Birmingham, England, UK

The submitted manuscript describes a study concluding that learning professionalism expectations is best achieved from a combination of both didactic teaching and the "informal curriculum". The manuscript is well written and the main strength of the study is that it was conducted across three universities which adds to the rigour of the study. The main drawback of the paper is that it does not include details of the questions asked of the students to lead them to their responses. This is particularly important regarding how the authors gauge the learning acquired from the "hidden curriculum" by the students. Consequently, it is difficult for the reader to ascertain the validity of the conclusions.

Have any limitations of the research been acknowledged?

Yes

Is the study design appropriate and does the work have academic merit?

Yes

Is the work clearly and accurately presented and does it cite the current literature?

Yes

If applicable, is the statistical analysis and its interpretation appropriate?

Yes

Are all the source data underlying the results available to ensure full reproducibility?

Yes

Are the conclusions drawn adequately supported by the results?

No

Are sufficient details of methods and analysis provided to allow replication by others?

No

Reviewer Expertise:

Professionalism

I confirm that I have read this submission and believe that I have an appropriate level of expertise to confirm that it is of an acceptable scientific standard, however I have significant reservations, as outlined above.
